# Pattern Discovery in Brain Imaging Genetics via SCCA Modeling with a Generic Non-convex Penalty

**DOI:** 10.1038/s41598-017-13930-y

**Published:** 2017-10-25

**Authors:** Lei Du, Kefei Liu, Xiaohui Yao, Jingwen Yan, Shannon L. Risacher, Junwei Han, Lei Guo, Andrew J. Saykin, Li Shen, Alzheimer’s Disease Neuroimaging Initiative, Michael W. Weiner, Paul Aisen, Ronald Petersen, Clifford R. Jack, William Jagust, John Q. Trojanowki, Arthur W. Toga, Laurel Beckett, Robert C. Green, John Morris, Leslie M. Shaw, Zaven Khachaturian, Greg Sorensen, Maria Carrillo, Lew Kuller, Marc Raichle, Steven Paul, Peter Davies, Howard Fillit, Franz Hefti, David Holtzman, M. Marcel Mesulam, William Potter, Peter Snyder, Adam Schwartz, Tom Montine, Ronald G. Thomas, Michael Donohue, Sarah Walter, Devon Gessert, Tamie Sather, Gus Jiminez, Archana B. Balasubramanian, Jennifer Mason, Iris Sim, Danielle Harvey, Matthew Bernstein, Nick Fox, Paul Thompson, Norbert Schuff, Charles DeCArli, Bret Borowski, Jeff Gunter, Matt Senjem, Prashanthi Vemuri, David Jones, Kejal Kantarci, Chad Ward, Robert A. Koeppe, Norm Foster, Eric M. Reiman, Kewei Chen, Chet Mathis, Susan Landau, Nigel J. Cairns, Erin Franklin, Lisa Taylor-Reinwald, Virginia Lee, Magdalena Korecka, Michal Figurski, Karen Crawford, Scott Neu, Tatiana M. Foroud, Steven Potkin, Kelley Faber, Sungeun Kim, Kwangsik Nho, Leon Thal, Neil Buckholtz, Marilyn Albert, Richard Frank, John Hsiao, Jeffrey Kaye, Joseph Quinn, Lisa Silbert, Betty Lind, Raina Carter, Sara Dolen, Lon S. Schneider, Sonia Pawluczyk, Mauricio Beccera, Liberty Teodoro, Bryan M. Spann, James Brewer, Helen Vanderswag, Adam Fleisher, Judith L. Heidebrink, Joanne L. Lord, Sara S. Mason, Colleen S. Albers, David Knopman, Kris Johnson, Rachelle S. Doody, Javier Villanueva-Meyer, Valory Pavlik, Victoria Shibley, Munir Chowdhury, Susan Rountree, Mimi Dang, Yaakov Stern, Lawrence S. Honig, Karen L. Bell, Beau Ances, Maria Carroll, Mary L. Creech, Erin Franklin, Mark A. Mintun, Stacy Schneider, Angela Oliver, Daniel Marson, David Geldmacher, Marissa Natelson Love, Randall Griffith, David Clark, John Brockington, Erik Roberson, Hillel Grossman, Effie Mitsis, Raj C. Shah, Leyla deToledo-Morrell, Ranjan Duara, Maria T. Greig-Custo, Warren Barker, Chiadi Onyike, Daniel D’Agostino, Stephanie Kielb, Martin Sadowski, Mohammed O. Sheikh, Anaztasia Ulysse, Mrunalini Gaikwad, P. Murali Doraiswamy, Jeffrey R. Petrella, Salvador Borges-Neto, Terence Z. Wong, Edward Coleman, Steven E. Arnold, Jason H. Karlawish, David A. Wolk, Christopher M. Clark, Charles D. Smith, Greg Jicha, Peter Hardy, Partha Sinha, Elizabeth Oates, Gary Conrad, Oscar L. Lopez, Mary Ann Oakley, Donna M. Simpson, Anton P. Porsteinsson, Bonnie S. Goldstein, Kim Martin, Kelly M. Makino, M. Saleem Ismail, Connie Brand, Adrian Preda, Dana Nguyen, Kyle Womack, Dana Mathews, Mary Quiceno, Allan I. Levey, James J. Lah, Janet S. Cellar, Jeffrey M. Burns, Russell H. Swerdlow, William M. Brooks, Liana Apostolova, Kathleen Tingus, Ellen Woo, Daniel H. S. Silverman, Po H. Lu, George Bartzokis, Neill R Graff-Radford, Francine Parfitt, Kim Poki-Walker, Martin R. Farlow, Ann Marie Hake, Brandy R. Matthews, Jared R. Brosch, Scott Herring, Christopher H. van Dyck, Richard E. Carson, Martha G. MacAvoy, Pradeep Varma, Howard Chertkow, Howard Bergman, Chris Hosein, Sandra Black, Bojana Stefanovic, Curtis Caldwell, Ging-Yuek Robin Hsiung, Benita Mudge, Vesna Sossi, Howard Feldman, Michele Assaly, Elizabeth Finger, Stephen Pasternack, Irina Rachisky, John Rogers, Dick Trost, Andrew Kertesz, Charles Bernick, Donna Munic, Emily Rogalski, Kristine Lipowski, Sandra Weintraub, Borna Bonakdarpour, Diana Kerwin, Chuang-Kuo Wu, Nancy Johnson, Carl Sadowsky, Teresa Villena, Raymond Scott Turner, Kathleen Johnson, Brigid Reynolds, Reisa A. Sperling, Keith A. Johnson, Gad Marshall, Jerome Yesavage, Joy L. Taylor, Barton Lane, Allyson Rosen, Jared Tinklenberg, Marwan N. Sabbagh, Christine M. Belden, Sandra A. Jacobson, Sherye A. Sirrel, Neil Kowall, Ronald Killiany, Andrew E. Budson, Alexander Norbash, Patricia Lynn Johnson, Thomas O. Obisesan, Saba Wolday, Joanne Allard, Alan Lerner, Paula Ogrocki, Curtis Tatsuoka, Parianne Fatica, Evan Fletcher, Pauline Maillard, John Olichney, Charles DeCarli, Owen Carmichael, Smita Kittur, Michael Borrie, T.-Y. Lee, Rob Bartha, Sterling Johnson, Sanjay Asthana, Cynthia M. Carlsson, Pierre Tariot, Anna Burke, Ann Marie Milliken, Nadira Trncic, Adam Fleisher, Stephanie Reeder, Vernice Bates, Horacio Capote, Michelle Rainka, Douglas W. Scharre, Maria Kataki, Brendan Kelly, Earl A. Zimmerman, Dzintra Celmins, Alice D. Brown, Godfrey D. Pearlson, Karen Blank, Karen Anderson, Laura A. Flashman, Marc Seltzer, Mary L. Hynes, Robert B. Santulli, Kaycee M. Sink, Leslie Gordineer, Jeff D. Williamson, Pradeep Garg, Franklin Watkins, Brian R. Ott, Geoffrey Tremont, Lori A. Daiello, Stephen Salloway, Paul Malloy, Stephen Correia, Howard J. Rosen, Bruce L. Miller, David Perry, Jacobo Mintzer, Kenneth Spicer, David Bachman, Nunzio Pomara, Raymundo Hernando, Antero Sarrael, Susan K. Schultz, Karen Ekstam Smith, Hristina Koleva, Ki Won Nam, Hyungsub Shim, Norman Relkin, Gloria Chaing, Michael Lin, Lisa Ravdin, Amanda Smith, Balebail Ashok Raj, Kristin Fargher

**Affiliations:** 1 School of Automation, Northwestern Polytechnical University, Xi’an, 710072 China; 2 Radiology and Imaging Sciences, Indiana University School of Medicine, Indianapolis, IN 46202 USA; 3 University of California, San Francisco, USA; 4 University of Southern California, Los Angeles, USA; 5 Mayo Clinic, Rochester, Minnesota USA; 6 University of California, Berkeley, Berkeley, USA; 7 University of Pennsylvania, Philadelphia, USA; 8 University of California, Davis, Davis, USA; 9 Brigham and Women’s Hospital/Harvard Medical School, Boston, USA; 10 Washington University St. Louis, St. Louis, USA; 11 Prevent Alzheimer’s Disease, 2020 Rockville, USA; 12 Siemens, Munich, Germany; 13 Alzheimer’s Association, Illinois, USA; 14 University of Pittsburgh, Pennsylvania, USA; 15 Cornell University, New York, USA; 16 Albert Einstein College of Medicine of Yeshiva University, New York, USA; 17 AD Drug Discovery Foundation, New York, USA; 18 Acumen Pharmaceuticals, California, USA; 19 Northwestern University, Illinois, USA; 20 National Institute of Mental Health, Maryland, USA; 21 Brown University, Rhode Island, USA; 22 Eli Lilly, Indiana, USA; 23 University of Washington, Washington, USA; 24 University of California, San Diego, California, USA; 25 University of London, London, UK; 26 University of California, Los Angeles, California, USA; 27 University of Michigan, Michigan, USA; 28 University of Utah, Utah, USA; 29 Banner Alzheimer’s Institute, Arizona, USA; 30 University of California, Irvine, California USA; 31 National Institute on Aging, Maryland, USA; 32 Johns Hopkins University, Maryland, USA; 33 Richard Frank Consulting, New Hampshire, USA; 34 Oregon Health and Science University, Oregon, USA; 35 Baylor College of Medicine, Texas, USA; 36 Columbia University Medical Center, New York, USA; 37 University of Alabama-Birmingham, Alabama, USA; 38 Mount Sinai School of Medicine, New York, USA; 39 Rush University Medical Center, Rush University, Illinois, USA; 40 Wien Center, Florida, USA; 41 New York University, New York, USA; 42 Duke University Medical Center, North Carolina, USA; 43 University of Kentucky, Kentucky, USA; 44 University of Rochester Medical Center, New York, USA; 45 University of Texas Southwestern Medical School, Texas, USA; 46 Emory University, Georgia, USA; 47 University of Kansas, Medical Center, Kansas, USA; 48 Mayo Clinic, Jacksonville, Florida USA; 49 Yale University School of Medicine, Connecticut, USA; 50 McGill University, Montreal-Jewish General Hospital, Quebec, Canada; 51 Sunnybrook Health Sciences, Ontario, Canada; 52 U.B.C. Clinic for AD & Related Disorders, British Columbia, Canada; 53 Cognitive Neurology-St. Joseph’s, Ontario, Canada; 54 Cleveland Clinic Lou Ruvo Center for Brain Health, Ohio, USA; 55 Premiere Research Inst (Palm Beach Neurology), Florida, USA; 56 Georgetown University Medical Center, Washington D.C, USA; 57 Stanford University, California, USA; 58 Banner Sun Health Research Institute, Arizona, USA; 59 Boston University, Massachusetts, USA; 60 Howard University, Washington D.C, USA; 61 Case Western Reserve University, Ohio, USA; 62 Neurological Care of CNY, New York, USA; 63 Parkwood Hospital, Pennsylvania, USA; 64 University of Wisconsin, Wisconsin, USA; 65 Dent Neurologic Institute, New York, USA; 66 Ohio State University, Ohio, USA; 67 Albany Medical College, New York, USA; 68 Hartford Hospital, Olin Neuropsychiatry Research Center, Connecticut, USA; 69 Dartmouth-Hitchcock Medical Center, New Hampshire, USA; 70 Wake Forest University Health Sciences, North Carolina, USA; 71 Rhode Island Hospital, Rhode Island, USA; 72 Butler Hospital, Rhode Island, USA; 73 Medical University South Carolina, Carolina, USA; 74 Nathan Kline Institute, New York, USA; 75 University of Iowa College of Medicine, Iowa, USA; 76 USF Health Byrd Alzheimer’s Institute, University of South Florida, Florida, USA

## Abstract

Brain imaging genetics intends to uncover associations between genetic markers and neuroimaging quantitative traits. Sparse canonical correlation analysis (SCCA) can discover bi-multivariate associations and select relevant features, and is becoming popular in imaging genetic studies. The L1-norm function is not only convex, but also singular at the origin, which is a necessary condition for sparsity. Thus most SCCA methods impose ${\ell }_{{\bf{1}}}$-norm onto the individual feature or the structure level of features to pursuit corresponding sparsity. However, the ${\ell }_{{\bf{1}}}$-norm penalty over-penalizes large coefficients and may incurs estimation bias. A number of non-convex penalties are proposed to reduce the estimation bias in regression tasks. But using them in SCCA remains largely unexplored. In this paper, we design a unified non-convex SCCA model, based on seven non-convex functions, for unbiased estimation and stable feature selection simultaneously. We also propose an efficient optimization algorithm. The proposed method obtains both higher correlation coefficients and better canonical loading patterns. Specifically, these SCCA methods with non-convex penalties discover a strong association between the *APOE* e4 rs429358 SNP and the hippocampus region of the brain. They both are Alzheimer’s disease related biomarkers, indicating the potential and power of the non-convex methods in brain imaging genetics.

## Introduction

By identifying the associations between genetic factors and brain imaging measurements, brain imaging genetics intends to model and understand how genetic factors influence the structure or function of human brain^1–14^. Both genetic biomarkers such as single nucleotide polymorphisms (SNPs), and brain imaging measurements such as imaging quantitative traits (QTs) are multivariate. To address this problem, bi-multivariate association models, such as multiple linear regression^15^, reduced rank regression^16–18^, parallel independent component analysis^19^, partial least squares regression^20,21^, canonical correlation analysis (CCA)^22^ and their sparsity-inducing variants^23^, have been widely used to uncover the joint effect of multiple SNPs on one or multiple QTs. Among them, SCCA (Sparse CCA), which can discover bi-multivariate relationships and extract relevant features, is becoming popular in brain imaging genetics.

The CCA technique has been introduced for several decades^24^. CCA can only perform well when the number of observations is larger than the combined feature number of the two views. Unfortunately, the problem usually is a *large-p-small-n* problem in the biomedical and biology studies. And it gets even worse because in CCA we are facing a *large-(p* + *q)-small-n* problem. In order to overcome this limitation, sparse CCA (SCCA)^25–36^ employs a sparsity inducing regularization term to select a small set of relevant features and has received increasing attention. The ${\ell }_{1}$-norm based SCCA method^25^ has gained great success for its sparsity pursuing capability. After that, there are many SCCA variants based on the ${\ell }_{1}$-norm. For examples, the fused lasso penalty imposes the ${\ell }_{1}$-norm onto the ordered pairwise features^25^, and the group lasso penalty imposes the ${\ell }_{1}$-norm onto the group of features^29,32^. Further, the graph lasso or the graph guided lasso can be viewed as imposing the ${\ell }_{1}$-norm onto the pairwise features defined by an undirected graph^29^.

However, the ${\ell }_{1}$-norm penalty shows the conflict of optimal prediction and consistent feature selection^37^. In penalized least squares modeling, Fan and Li^38^ showed that a good penalty function should meet three properties. First, the penalty function should be singular at the origin to produce sparse results. Second, it should produce continuous models for stable model selection, and third, the penalty function should not penalize large coefficients to avoid estimation bias. The ${\ell }_{1}$-norm penalty is successful in feature selection because it is singular at the origin. On the contrary, the ${\ell }_{1}$-norm penalty over-penalizes large coefficients, and thus it may be suboptimal with respect to the estimation risk^39,40^. The ${\ell }_{0}$-norm function which only involves the number of nonzero features is an ideal sparsity-inducing penalty. However, it is neither convex nor continuous, and thus solving ${\ell }_{0}$-norm constrained problem is NP-hard^41^.

A number of non-convex penalties are proposed as the surrogate of the ${\ell }_{0}$-norm to handle this issue. These penalties includes the ${\ell }_{\gamma }$-norm (0 < *γ* < 1) penalty^42^, the Geman penalty^43^, the Smoothly Clipped Absolute Deviation (SCAD) penalty^38^, the Laplace penalty^44^, the Minimax Concave Penalty (MCP)^45^, the Exponential-Type Penalty (ETP)^46^ and the Logarithm penalty^47^. These non-convex functions have attractive theoretical properties for they all are singular at the origin and leave those larger coefficients unpenalized. Though they have gained great success in generalized linear models (GLMs), it is an unexplored topic to apply them to the SCCA models for achieving sparsity and unbiased prediction simultaneously.

Therefore, it is essential and of great interest to investigate performances of various SCCA models based on these non-convex penalties. A major challenge of non-convex function is the computational complexity. The local quadratic approximation (LQA) technique is introduced to solve the SCAD penalizing problem^38^. LQA approximates the objective by a locally quadratic expression which can be solved like a ridge constrained problem. Inspired by this, in this paper, we propose a generic non-convex SCCA models with these non-convex penalties, and propose a unified optimization algorithm based on the LQA technique and the Alternate Convex Search (ACS) method^48^. Using both synthetic data and real imaging genetic data, the experimental results show that with appropriate parameters, the non-convex SCCA methods have better performance on both canonical loading patterns and correlation coefficients estimation than the ${\ell }_{1}$-norm based SCCA methods.

## Methods

Throughout this paper, scalars are denoted as italic letters, column vectors as boldface lowercase letters, and matrices as boldface capitals. The $\Vert {\bf{u}}\Vert$ denotes the Euclidean norm of a vector **u**.

### Preliminaries

#### Sparse Canonical Correlation Analysis (SCCA)

Let ${\bf{X}}\in { {\mathcal R} }^{n\times p}$ be a matrix representing the SNP biomarkers data, where *n* is the number of participants and *p* is the number of SNPs. Let ${\bf{Y}}\in { {\mathcal R} }^{n\times q}$ be the QT data with *q* being the number of imaging measurements. A typical SCCA model is defined as

1 $$\mathop{{\rm{\min }}}\limits_{{\bf{u}},{\bf{v}}}-{{\bf{u}}}^{{\rm{{\rm T}}}}{{\bf{X}}}^{{\rm{{\rm T}}}}{\bf{Y}}{\bf{v}}$$

$$s\mathrm{.}t\mathrm{.\ \ }{\Vert {\bf{X}}{\bf{u}}\Vert }^{2}\le 1,{\Vert {\bf{Y}}{\bf{v}}\Vert }^{2}\le 1,{\rm{\Omega }}({\bf{u}})\le {c}_{1},{\rm{\Omega }}({\bf{v}})\le {c}_{2},$$

where **Xu** and **Yv** are the canonical variables, **u** and **v** are the corresponding canonical vectors we desire to estimate, and *c*_1_, *c*_2_ are the tuning parameters that control the sparsity level of the solution. The penalty function could be the ${\ell }_{1}$-norm penalty, or its variants such as the fused lasso, group lasso and graph lasso^25,27,29,32,34^.

#### Non-convex Penalty Functions for SCCA

**Figure 1:**
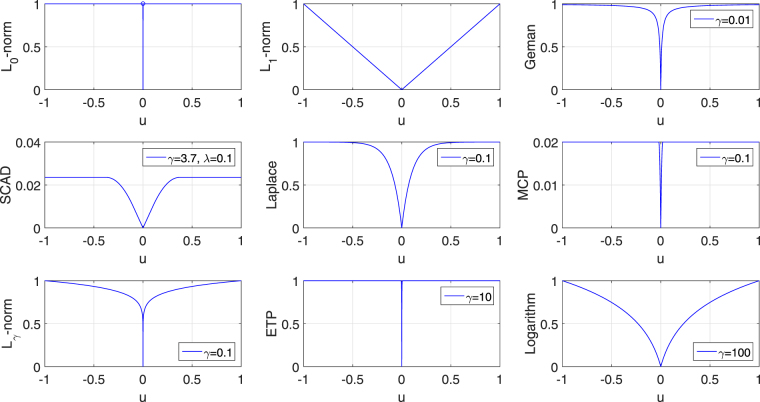
Illustration of the ${\ell }_{0}$, ${\ell }_{1}$ and seven non-convex functions. All the non-convex penalty functions share two common properties: They are singular at origin, concave and monotonically decreasing on (−∞,0], and concave and monotonically increasing on [0,∞).

In this paper, we investigate seven non-convex surrogate penalties of ${\ell }_{0}$-norm in the SCCA model. They are singular at the origin, which is essential to achieve sparsity in the solution. And they do not overly penalize large coefficients. In order to facilitate a unified description, we denote the non-convex penalty as

2 $${{\rm{\Omega }}}_{{\rm{nc}}}({\bf{u}})=\sum _{i=1}^{p}{P}_{\lambda ,\gamma }(|{u}_{i}|),$$

where *λ* and *γ* are nonnegative parameters, and *P*_*λ*,*γ*_(|*u*_*i*_|) is a non-convex function. We absorb *λ* into the penalty because it cannot be decoupled from several penalties, such as the SCAD function^38^. We here have seven penalties and they are described in Table 1 and visualized in Fig. 1, where for clarity we have dropped the subscript *i* in *u*_*i*_. There is a sharp point at the origin for each of them, indicating that they are singular at the origin. This is essential to achieve sparseness in the solution. Besides, these curves are concave in |*u*_*i*_| and monotonically decreasing on (−∞, 0], and monotonically increasing on [0, ∞). Therefore, though these penalties are not convex, they are piecewise continuously differentiable and their supergradients exist on both (−∞, 0] and [0, ∞)^49^. Table 1 also shows their supergradients *P*′_*λ*,*γ*_(|*u*_*i*_|) with respect to |*u*_*i*_|.

**Table 1:** The seven non-convex penalty functions and their supergradients.

Penalty Name	Function (*P*_*λ*,*γ*_(|*u*|))	Supergradient (*P*′*λ*,*γ*(|*u*|))
${\ell }_{\gamma }$-norm^42^	*λ*|*u*|^*γ*^	$$\{\begin{array}{ll}\infty , & u=\mathrm{0,}\\ \lambda \gamma |u{|}^{\gamma -1}, & |u| > 0.\end{array}$$
Geman^43^	$\frac{\lambda |u|}{|u|+\gamma }$	$\frac{\lambda \gamma }{{(|u|+\gamma )}^{2}}$
SCAD^38^	$$\{\begin{array}{ll}\lambda |u|, & |u|\le \lambda \\ \frac{-|u{|}^{2}+2\gamma \lambda |u|-{\lambda }^{2}}{\mathrm{2(}\gamma -\mathrm{1)}}, & \lambda \le |u|\le \gamma \lambda \\ \frac{{\lambda }^{2}(\gamma +\mathrm{1)}}{2}, & |u|\ge \gamma \lambda \mathrm{.}\end{array}$$	$$\{\begin{array}{ll}\lambda , & |u|\le \lambda \\ \frac{\gamma \lambda -|u|}{\gamma -1}, & \lambda \le |u|\le \gamma \lambda \\ \mathrm{0,} & |u|\ge \gamma \lambda \mathrm{.}\end{array}$$
Laplace^44^	$\lambda (1-\exp (-\frac{|u|}{\gamma }))$	$\frac{\lambda }{\gamma }\exp (-\frac{|u|}{\gamma })$
MCP^45^	$$\{\begin{array}{ll}\lambda |u|-\frac{|u{|}^{2}}{2\gamma }, & |u|\le \gamma \lambda \\ \frac{1}{2}\gamma {\lambda }^{2}, & |u|\ge \gamma \lambda \mathrm{.}\end{array}$$	$$\{\begin{array}{ll}\lambda -\frac{|u|}{\gamma }, & |u|\le \gamma \lambda \\ \mathrm{0,} & |u|\ge \gamma \lambda \mathrm{.}\end{array}$$
ETP^46^	$\frac{\lambda }{1-\exp (-\gamma )}(1-\exp (-\gamma |u|))$	$\frac{\lambda \gamma }{1-\exp (-\gamma )}\exp (-\gamma |u|)$
Logarithm^47^	$\frac{\lambda }{\mathrm{log}(\gamma +1)}\,\mathrm{log}(\gamma |u|+1)$	$\frac{\lambda \gamma }{(\gamma |u|+1)\mathrm{log}(\gamma +1)}$

### The Proposed Non-convex SCCA Model and Optimization Algorithm

Replacing the ${\ell }_{1}$-norm constraints in the SCCA model, we define the unified non-convex SCCA model as follows

3 $$\mathop{{\rm{\min }}}\limits_{{\bf{u}},{\bf{v}}}-{{\bf{u}}}^{{\rm{{\rm T}}}}{{\bf{X}}}^{{\rm{{\rm T}}}}{\bf{Y}}{\bf{v}}+{{\rm{\Omega }}}_{{\rm{nc}}}({\bf{u}})+{{\rm{\Omega }}}_{{\rm{nc}}}({\bf{v}})$$

$$s\mathrm{.}t\mathrm{.\ \ }{\Vert {\bf{X}}{\bf{u}}\Vert }^{2}\le \mathrm{1,}\,{\Vert {\bf{Y}}{\bf{v}}\Vert }^{2}\le \mathrm{1,}$$

where Ω_nc_(**u**) and Ω_nc_(**v**) can be any of the non-convex functions listed in Table 1.

To solve the non-convex SCCA problem, we use the Lagrangian method,

4 $$ {\mathcal L} {\boldsymbol{(}}{\bf{u}}{\boldsymbol{,}}{\bf{v}}{\boldsymbol{)}}=-{{\bf{u}}}^{{\rm{{\rm T}}}}{{\bf{X}}}^{{\rm{{\rm T}}}}{\bf{Y}}{\bf{v}}+{{\rm{\Omega }}}_{{\rm{nc}}}({\bf{u}})+{{\rm{\Omega }}}_{{\rm{nc}}}({\bf{v}})+\frac{{\alpha }_{1}}{2}({\Vert {\bf{X}}{\bf{u}}\Vert }^{2}-1)+\frac{{\alpha }_{2}}{2}({\Vert {\bf{Y}}{\bf{v}}\Vert }^{2}-1),$$

which is equivalent to

5 $$ {\mathcal L} {\boldsymbol{(}}{\bf{u}}{\boldsymbol{,}}{\bf{v}}{\boldsymbol{)}}=-{{\bf{u}}}^{{\rm{{\rm T}}}}{{\bf{X}}}^{{\rm{{\rm T}}}}{\bf{Y}}{\bf{v}}+{{\rm{\Omega }}}_{{\rm{nc}}}({\bf{u}})+{{\rm{\Omega }}}_{{\rm{nc}}}({\bf{v}})+\frac{{\alpha }_{1}}{2}{\Vert {\bf{X}}{\bf{u}}\Vert }^{2}+\frac{{\alpha }_{2}}{2}{\Vert {\bf{Y}}{\bf{v}}\Vert }^{2}$$

from the point of view of optimization. *α*_1_, *α*_2_, *λ*_1_, *λ*_2_ and *γ* are nonnegative tuning parameters. Next we will show how to solve this non-convex problem.

The first term −**u** ^Τ^ **X** ^Τ^ **Yv** on the right of equation (5) is biconvex in **u** and **v**. ${\Vert {\bf{X}}{\bf{u}}\Vert }^{2}$ is convex in **u**, and ${\Vert {\bf{Y}}{\bf{v}}\Vert }^{2}$ is convex in **v**. It remains to approximate both Ω_nc_(**u**) and Ω_nc_(**v**) and transform them into convex ones.

The local quadratic approximation (LQA) technique was introduced to quadratically expresses the SCAD penalty^38^. Based on LQA, we here show how to represent these non-convex penalties in a unified way. First, we have the first-order Taylor expansion of ${P}_{{\lambda }_{1},\gamma }(\sqrt{\mu })$ at *μ*_0_ *P*_*λ*,*γ*_((*μ*)^1/2^) at *μ*_0_

6 $${P}_{\lambda ,\gamma }(\sqrt{\mu })\approx {P}_{\lambda ,\gamma }(\sqrt{{\mu }_{0}})+{P^{\prime} }_{\lambda ,\gamma }(\sqrt{{\mu }_{0}})\frac{1}{2\sqrt{{\mu }_{0}}}(\mu -{\mu }_{0}),$$

where *μ*_0_ and *μ* are neighbors, e.g., the estimates at two successive iterations during optimization. Substituting $\mu ={u}_{i}^{2}$ and ${\mu }_{0}={({u}_{i}^{t})}^{2}$ into (6), we have

7 $${P}_{\lambda ,\gamma }(|{u}_{i}|)\approx {P}_{\lambda ,\gamma }(|{u}_{i}^{t}|)+{P^{\prime} }_{\lambda ,\gamma }(|{u}_{i}^{t}|)\frac{1}{\mathrm{2|}{u}_{i}^{t}|}({u}_{i}^{2}-{({u}_{i}^{t})}^{2})$$

with ${P^{\prime} }_{\lambda ,\gamma }(|{u}_{i}^{t}|)$ being the supergradient of ${P}_{\lambda ,\gamma }(|{u}_{i}^{t}|)$ (as shown in Table 1) at $|{u}_{i}^{t}|$.

Then we obtain a quadratic approximation to Ω_nc_(**u**):

8 $${{\rm{\Omega }}}_{{\rm{nc}}}({\bf{u}})=\sum _{i\mathrm{=1}}^{p}{P}_{\lambda ,\gamma }(|{u}_{i}|)\approx \sum _{i\mathrm{=1}}^{p}\frac{{P^{\prime} }_{\lambda ,\gamma }(|{u}_{i}^{t}|)}{\mathrm{2|}{u}_{i}^{t}|}{u}_{i}^{2}+{C}_{{\bf{u}}},$$

where

$${C}_{{\bf{u}}}=\sum _{i\mathrm{=1}}^{p}[{P}_{\lambda ,\gamma }(|{u}_{i}^{t}|)-\frac{1}{2}{P^{\prime} }_{\lambda ,\gamma }(|{u}_{i}^{t}|)|{u}_{i}^{t}|]$$

is not a function of **u** and thus will not contribute to the optimization.

In a similar way, we can construct a quadratic approximation to Ω_nc_(**v**)

9 $${{\rm{\Omega }}}_{{\rm{nc}}}({\bf{v}})=\sum _{j\mathrm{=1}}^{q}{P}_{\lambda ,\gamma }(|{v}_{j}|)\approx \sum _{j\mathrm{=1}}^{q}\frac{{P^{\prime} }_{\lambda ,\gamma }(|{v}_{j}^{t}|)}{\mathrm{2|}{v}_{j}^{t}\mathrm{|}}{v}_{j}^{2}+{C}_{{\bf{v}}},$$

where

$${C}_{{\bf{v}}}=\sum _{j\mathrm{=1}}^{q}[{P}_{\lambda ,\gamma }(|{v}_{j}^{t}|)-\frac{1}{2}{P^{\prime} }_{\lambda ,\gamma }(|{v}_{j}^{t}|)|{v}_{j}^{t}|]$$

is not a function of **v** and makes no contribute towards the optimization.

Denote the estimates of **u** and **v** in the *t*-th iteration as **u** ^*t*^ and **v** ^*t*^, respectively. To update the estimates of **u** and **v** in the (*t* + 1)-th iteration, we substitute the approximate functions of Ω_nc_(**u**) and Ω_nc_(**v**) in equations (8) and (9) into ${\mathcal L} {\boldsymbol{(}}{\bf{u}}{\boldsymbol{,}}{\bf{v}}{\boldsymbol{)}}$ in 5, and solve the resultant approximate version of the original problem:

10 $$\begin{array}{rcl}{\rm{\arg }}\,{\rm{\min }}\, {\mathcal L} {\boldsymbol{(}}{\bf{u}}{\boldsymbol{,}}{\bf{v}}{\boldsymbol{)}} & = & {\rm{\arg }}\,{\rm{\min }}\,-{{\bf{u}}}^{{\rm{{\rm T}}}}{{\bf{X}}}^{{\rm{{\rm T}}}}{\bf{Y}}{\bf{v}}+\sum _{i\mathrm{=1}}^{p}\frac{{P^{\prime} }_{{\lambda }_{1},\gamma }(|{u}_{i}^{t}|)}{\mathrm{2|}{u}_{i}^{t}|}{u}_{i}^{2}\\  &  & +\,\sum _{j\mathrm{=1}}^{q}\frac{{P^{\prime} }_{{\lambda }_{1},\gamma }(|{v}_{j}^{t}|)}{\mathrm{2|}{v}_{j}^{t}|}{v}_{j}^{2}+\frac{{\alpha }_{1}}{2}||{\bf{X}}{\bf{u}}{||}^{2}+\frac{{\alpha }_{2}}{2}||{\bf{Y}}{\bf{v}}{||}^{2}\end{array}$$

Obviously, the equation (10) is a quadratical expression, and is biconvex in **u** and **v**. This means it is convex in terms of **u** given **v**, and vice versa. Then according to the alternate convex search (ACS) method which is designed to solve biconvex problems^48^, the (*t* + 1)-th estimation of **u** and **v** can be calculated via

11 $$\begin{array}{rcl}{{\bf{u}}}^{t+1} & = & {\rm{\arg }}\,\mathop{{\rm{\min }}}\limits_{{\bf{u}}}-{{\bf{u}}}^{{\rm{{\rm T}}}}{{\bf{X}}}^{{\rm{{\rm T}}}}{\bf{Y}}{{\bf{v}}}^{t}+\sum _{i\mathrm{=1}}^{p}\frac{{P^{\prime} }_{{\lambda }_{1},\gamma }(|{u}_{i}^{t}|)}{\mathrm{2|}{u}_{i}^{t}|}{u}_{i}^{2}+\frac{{\alpha }_{1}}{2}||{\bf{X}}{\bf{u}}{||}^{2},\\ {{\bf{v}}}^{t+1} & = & {\rm{\arg }}\,\mathop{{\rm{\min }}}\limits_{{\bf{v}}}-{({{\bf{u}}}^{t+1})}^{{\rm{{\rm T}}}}{{\bf{X}}}^{{\rm{{\rm T}}}}{\bf{Y}}{\bf{v}}+\sum _{j\mathrm{=1}}^{q}\frac{{P^{\prime} }_{{\lambda }_{2},\gamma }(|{v}_{j}^{t}|)}{\mathrm{2|}{v}_{j}^{t}|}{v}_{j}^{2}+\frac{{\alpha }_{2}}{2}||{\bf{Y}}{\bf{v}}{||}^{2}\mathrm{.}\end{array}$$

Both equations above are quadratic, and thus their closed-form solutions exist. Taking the partial derivative of ${\mathcal L} {\boldsymbol{(}}{\bf{u}}{\boldsymbol{,}}{\bf{v}}{\boldsymbol{)}}$ in (5) with respect to **u** and **v** and setting the results to zero, we have

12 $${\bf{0}}\in -{{\bf{X}}}^{{\rm{{\rm T}}}}{\bf{Y}}{\bf{v}}+({{\bf{D}}}_{1}^{t}+{\alpha }_{1}{{\bf{X}}}^{{\rm{{\rm T}}}}{\bf{X}}){\bf{u}},$$

13 $${\bf{0}}\in -{{\bf{Y}}}^{{\rm{{\rm T}}}}{\bf{X}}{\bf{u}}+({{\bf{D}}}_{2}^{t}+{\alpha }_{2}{{\bf{Y}}}^{{\rm{{\rm T}}}}{\bf{Y}}){\bf{v}},$$

where ${{\bf{D}}}_{1}^{t}$ is a diagonal matrix with the *i*-th diagonal entry as $\frac{{P^{\prime} }_{{\lambda }_{1},\gamma }(|{u}_{i}^{t}|)}{|{u}_{i}^{t}|}$ (*i*∈[1, *p*]). It can be calculated by taking the partial derivative of equation (7) with respect to *u*_*i*_. ${{\bf{D}}}_{2}^{t}$ is also a diagonal matrix with the *j*-th diagonal entry as $\frac{{P^{\prime} }_{{\lambda }_{1},\gamma }(|{v}_{j}^{t}|)}{|{v}_{j}^{t}|}$ (*j*∈[1, *q*]), and can be computed similarly. However, the *i*-th element of ${{\bf{D}}}_{1}^{t}$ does not exist if ${u}_{i}^{t}=0$. According to perturbed version of LQA^50^, we address this by adding a slightly perturbed term. Then the *i*-th element of ${{\bf{D}}}_{1}^{t}$ is

14 $${{\bf{D}}}_{1}^{t}(i,i)=\frac{{P^{\prime} }_{{\lambda }_{1},\gamma }(|{u}_{i}|)}{|{u}_{i}|+\zeta }$$

where *ζ* is a tiny positive number. Hunter and Li^50^ showed that this modification guarantees optimizing the equation (11). Then we have the updating expressions at the (*t* + 1)-th iteration

15 $${{\bf{u}}}^{t+1}=({{\bf{D}}}_{1}^{t}+{\alpha }_{1}{{\bf{X}}}^{{\rm{{\rm T}}}}{\bf{X}}{)}^{-1}{{\bf{X}}}^{{\rm{{\rm T}}}}{\bf{Y}}{{\bf{v}}}^{t},$$

16 $${{\bf{v}}}^{t+1}=({{\bf{D}}}_{2}^{t}+{\alpha }_{2}{{\bf{Y}}}^{{\rm{{\rm T}}}}{\bf{Y}}{)}^{-1}{{\bf{Y}}}^{{\rm{{\rm T}}}}{\bf{X}}{{\bf{u}}}^{t+1}\mathrm{.}$$

We alternate between the above two equations to graduate refine the estimates for **u** and **v** until convergence. The pseudo code of the non-convex SCCA algorithm is described in Algorithm 1.
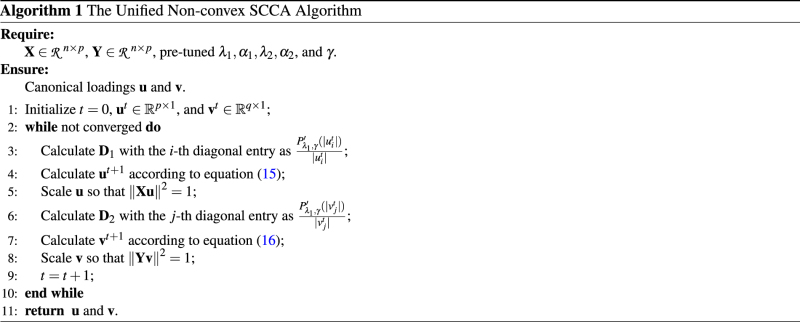


### Computational Analysis

In Algorithm 1, Step 3 and Step 6 are linear in the dimension of **u** and **v**, and are easy to compute. Step 4 and Step 7 are the critical steps of proposed algorithm. Since we have closed-form updating expressions, they can be calculated via solving a system of linear equations with quadratic complexity which avoids computing the matrix inverse with cubic complexity. Step 5 and 8 are the re-scale step and very easy to calculate. Therefore, the whole algorithm is efficient.

### Data Availability

The synthetic data sets generated in this work are available from the corresponding authors’ web sites, http://www.escience.cn/people/dulei/code.html and http://www.iu.edu/ shenlab/tools/ncscca/. The real data set is publicly available in the Alzheimer’s Disease Neuroimaging Initiative (ADNI) database repository, http://adni.loni.usc.edu.

## Experiments and Datasets

### Data Description

#### Synthetic Dataset

**Figure 2:**
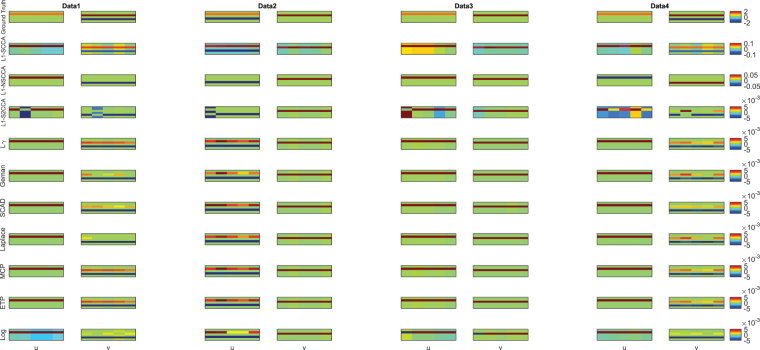
Canonical loadings estimated on four synthetic data sets. The first column shows results for Data1, and the second column is for Data2, and so forth. The first row is the ground truth, and each remaining one corresponds to an SCCA method: (1) Ground Truth. (2) L1-SCCA. (3) L1-NSCCA. (4) L1-S2CCA. (5) ${\ell }_{\gamma }$-norm and so forth. For each data set and each method, the estimated weights of **u** is shown on the left panel, and **v** is on the right. In each individual heat map, the x-axis indicates the indices of elements in u or v; the y-axis indicates the indices of the cross-validation folds.

There are four data sets with sparse true signals for both **u** and **v**, i.e., only a small subset of features are nonzero. The number of features of both ***u*** and ***v*** are larger than the observations to simulate a *large-(p* + *q)-small-n* task. The generating process is as follows. We first generate **u** and **v** with most feature being zero. After that, the latent variable z is constructed from Gaussian distribution *N*(**0**, ***I***_*n* × *n*_). Then we create the data **X** from ${{\bf{x}}}_{i}\sim N({z}_{i}{\bf{u}},{\sum }_{x})$ and data ${{\bf{y}}}_{i}\sim N({z}_{i}{\bf{v}},{\sum }_{y})$, where (∑_*x*_)_*jk*_ = exp(−|*u*_*j*_ − *u*_*k*_|) and (∑_*y*_)_*jk*_ = exp(−|*v*_*j*_ − *v*_*k*_|). The first three sets have 250 features for **u** and 600 ones for **v**, but they have different correlation coefficients. There are 500 features and 900 features in **u** and **v** respectively for the last data set. We show the true signal of every data set in Fig. 2 (top row).

#### Real Neuroimaging Genetics Dataset

Data used in the preparation of this article were obtained from the ADNI database (adni.loni.usc.edu). The ADNI was launched in 2003 as a public-private partnership by the National Institute on Aging (NIA), the National Institute of Biomedical Imaging and Bioengineering (NIBIB), the Food and Drug Administration (FDA) etc, led by Principal Investigator Michael W. Weiner, MD. The primary goal of ADNI has been to test whether serial magnetic resonance imaging (MRI), positron emission tomography (PET), other biological markers, and clinical and neuropsychological assessment can be combined to measure the progression of mild cognitive impairment (MCI) and early Alzheimer’s disease (AD). For up-to-date information, see www.adni-info.org. The study protocols were approved by the Institutional Review Boards of all participating centers (Northwestern Polytechnical University, Indiana University and ADNI (A complete list of ADNI sites is available at http://www.adni-info.org/)) and written informed consent was obtained from all participants or authorized representatives. All the analyses were performed on the de-identified ADNI data, and were determined by Indiana University Human Subjects Office as IU IRB Review Not Required.

The real neuroimaging genetics dataset were collected from 743 participants, and the details was presented in Table 2. There were 163 candidate SNP biomarkers from the AD-risk genes, e.g., *APOE*, in the genotyping data. The structural MRI scans were processed with voxel-based morphometry (VBM) in SPM8^51,52^. Briefly, scans were aligned to a T1-weighted template image, segmented into gray matter (GM), white matter (WM) and cerebrospinal fluid (CSF) maps, normalized to MNI space, and smoothed with an 8mm FWHM kernel. We subsampled the whole brain and generated 465 voxels spanning the whole brain ROIs. The regression technique was employed to remove the effects of the baseline age, gender, education, and handedness for these VBM measures. The aim of this study is to evaluate the correlation between the SNPs and the VBM measures, and further identify which SNPs and ROIs are associated.

**Table 2:** Participant characteristics.

	HC	MCI	AD
Num	204	363	176
Gender(M/F)	111/93	235/128	95/81
Handedness(R/L)	190/14	329/34	166/10
Age(mean ± std)	76.07 ± 4.99	74.88 ± 7.37	75.60 ± 7.50
Education(mean ± std)	16.15 ± 2.73	15.72 ± 2.30	14.84 ± 3.12

### Experimental Setup

#### Benchmarks

In this paper, we are mainly interested in whether these non-convex SCCA methods could enhance the performance of ${\ell }_{1}$-SCCA method based on our motivation. It is reasonable to employ the ${\ell }_{1}$-norm based methods in comparison. Therefore, the structure-aware SCCA methods such as^28,29,32,34^ are not contained here as benchmark. Based on different mathematical techniques, there are three different ${\ell }_{1}$-SCCA algorithms. They are the singular value decomposition based method^25^, the primal-dual based method^29^ and the LQA based method^32^. Though the latter two are proposed for capturing group or network structure, they can be easily reformulated to the ${\ell }_{1}$-norm constrained methods, such as setting the parameters associated with the structure penalty to zero^29^. Therefore, to make the comparison fair and convincing, we choose all of them as benchmarks. With a slight abuse of notation, we use the penalty name to refer a non-convex SCCA method, e.g. ETP for ETP based SCCA method. For the ${\ell }_{1}$-norm based methods, we call them L1-SCCA^25^, L1-S2CCA^32^, and L1-NSCCA^29^.

#### Parameter Tuning

There are four parameters *λ*_*i*_(*i* = 1, 2) and *α*_*i*_(*i* = 1, 2) associated with the non-convex SCCA methods, and one pivotal parameter *γ*. According to their equations, these non-convex penalties can approximate the ${\ell }_{0}$-norm by providing an appropriate *γ*. In this situation, the *λ*_*i*_ and *α*_*i*_ play a very weak role because theoretically the ${\ell }_{0}$-norm penalized problem does not rely on the parameters. Based on this consideration, we here only tune the *γ* other than tuning *λ*_*i*_ and *α*_*i*_ by a grid search strategy. This reduces the time consumption dramatically but does not affect the performance significantly. Further, we observe that two *γ*'s perform similarly if they are not significantly different. Thus the tuning range of *γ* is not continuous. Besides, we set *γ* = 3.7 for SCAD penalty since^38^ suggested that this is a very reasonable choice. The details of tuning range for each penalty are contained in Table 3. For *λ*_*i*_ and *α*_*i*_, we simply set them to 1 in this study.

**Table 3:** The searching range of optimal *γ* for each non-convex penalty.

	${\ell }_{\gamma }$-norm	SCAD	Geman, Laplace, MCP	ETP, Log
Range of *γ*	0.1, 0.2, 0.3	3.7	0.1, 0.01, 0.001	10, 100, 1000

#### Termination Criterion

We use ${{\rm{\max }}}_{i}|{u}_{i}^{t+1}-{u}_{i}^{t}|\le \varepsilon$ and ${{\rm{\max }}}_{j}|{v}_{j}^{t+1}-{v}_{j}^{t}|\le \varepsilon$ as the termination condition for Algorithm 1, where *ε* is the user defined error bound. In this study, we set *ε* = 10^−5^ according to experiments. All methods use the same setup, i.e., the same partition of the five-fold cross-validation, running on the same platform.

### Results on Synthetic Data

Figure 2 shows the heat maps of canonical loadings estimated from all SCCA methods, where each row corresponds to an experimental method. We clearly observe that the non-convex SCCA methods and L1-SCCA correctly identify the identical signal positions to the ground truth across four data sets. Besides true signals, L1-SCCA introduces several undesired signals which makes it be inferior to our methods. As a contrast, L1-NSCCA finds out an incomplete proportion of the ground truth, and L1-S2CCA performs unstably as it fails on some folds. Moreover, we also prioritize these methods using the AUC (area under ROC) criterion in Table 4, where a higher value indicates a better performance. The results exhibit that the non-convex SCCA methods have the highest score at almost every case. L1-SCCA scores similarly to the proposed methods, but later we can see it pays the price at a reduced prediction ability. Table 5 presents the estimated correlation coefficients on both training and testing data, where the best values are shown in boldface. The proposed SCCA methods alternatively gain the best value, and the Log method wins out for the most times. This demonstrates that the non-convex methods outperform ${\ell }_{1}$-norm based SCCA methods in terms of the prediction power. In summary, the proposed methods identify accurate and sparse canonical loading patterns and obtain high correlation coefficients simultaneously, while those ${\ell }_{1}$-norm based SCCA methods cannot.

**Table 4:** Performance comparison on synthetic data sets. The AUC (area under the curve) values (mean ± std) of estimated canonical loadings **u** and **v**.

	u				v			
	Data1	Data2	Data3	Data4	Data1	Data2	Data3	Data4
L1-SCCA	1.00 ± 0.00	0.75 ± 0.00	1.00 ± 0.00	1.00 ± 0.00	1.00 ± 0.00	0.74 ± 0.10	1.00 ± 0.00	1.00 ± 0.00
L1-S2CCA	1.00 ± 0.00	0.38 ± 0.00	1.00 ± 0.00	1.00 ± 0.00	0.75 ± 0.00	0.75 ± 0.00	1.00 ± 0.00	0.75 ± 0.00
L1-NSCCA	0.80 ± 0.45	0.30 ± 0.41	0.80 ± 0.45	0.40 ± 0.55	1.00 ± 0.00	0.65 ± 0.15	1.00 ± 0.00	0.80 ± 0.27
${\ell }_{\gamma }$-norm	1.00 ± 0.00	0.75 ± 0.00	1.00 ± 0.00	1.00 ± 0.00	1.00 ± 0.00	0.76 ± 0.04	1.00 ± 0.00	1.00 ± 0.00
Geman	1.00 ± 0.00	0.75 ± 0.00	1.00 ± 0.00	1.00 ± 0.00	1.00 ± 0.00	0.74 ± 0.01	1.00 ± 0.00	1.00 ± 0.00
SCAD	1.00 ± 0.00	0.75 ± 0.00	1.00 ± 0.00	1.00 ± 0.00	1.00 ± 0.00	0.74 ± 0.02	1.00 ± 0.00	1.00 ± 0.00
Laplace	1.00 ± 0.00	0.75 ± 0.00	1.00 ± 0.00	1.00 ± 0.00	1.00 ± 0.00	0.75 ± 0.02	1.00 ± 0.00	1.00 ± 0.00
MCP	1.00 ± 0.00	0.75 ± 0.00	1.00 ± 0.00	1.00 ± 0.00	1.00 ± 0.00	0.76 ± 0.04	1.00 ± 0.00	1.00 ± 0.00
ETP	1.00 ± 0.00	0.75 ± 0.00	1.00 ± 0.00	1.00 ± 0.00	1.00 ± 0.00	0.76 ± 0.04	1.00 ± 0.00	1.00 ± 0.00
Log	1.00 ± 0.00	0.75 ± 0.00	1.00 ± 0.00	1.00 ± 0.00	1.00 ± 0.00	0.75 ± 0.02	1.00 ± 0.00	1.00 ± 0.00

**Table 5:** Training and testing correlation coefficients (mean ± std) of 5-fold cross-validation synthetic data sets. The best values are shown in boldface.

	Training				Testing			
	data1	data2	data3	data4	data1	data2	data3	data4
L1-SCCA	0.65 ± 0.03	0.83 ± 0.03	0.65 ± 0.05	0.66 ± 0.04	0.59 ± 0.14	0.82 ± 0.05	0.59 ± 0.25	0.62 ± 0.08
L1-S2CCA	0.51 ± 0.25	0.67 ± 0.30	0.63 ± 0.28	0.32 ± 0.15	0.55 ± 0.23	0.68 ± 0.28	0.53 ± 0.29	0.24 ± 0.20
L1-NSCCA	0.62 ± 0.04	0.80 ± 0.01	0.75 ± 0.01	0.65 ± 0.02	0.61 ± 0.17	0.80 ± 0.04	0.73 ± 0.13	0.65 ± 0.10
${\ell }_{\gamma }$-norm	0.62 ± 0.04	**0.83** ± **0.01**	0.75 ± 0.01	0.65 ± 0.02	0.61 ± 0.17	**0.84** ± **0.02**	0.73 ± 0.13	0.66 ± 0.10
Geman	0.62 ± 0.04	**0.83** ± **0.01**	0.75 ± 0.01	0.65 ± 0.02	0.62 ± 0.17	0.83 ± 0.02	0.72 ± 0.13	0.66 ± 0.10
SCAD	0.62 ± 0.04	**0.83** ± **0.01**	0.75 ± 0.01	0.65 ± 0.03	0.61 ± 0.17	**0.84** ± **0.02**	0.73 ± 0.13	0.66 ± 0.10
Laplace	0.62 ± 0.04	**0.83** ± **0.01**	0.75 ± 0.01	0.65 ± 0.02	0.61 ± 0.17	0.83 ± 0.02	0.73 ± 0.13	0.66 ± 0.10
MCP	0.62 ± 0.04	**0.83** ± **0.01**	0.75 ± 0.01	0.65 ± 0.02	0.61 ± 0.17	**0.84** ± **0.02**	0.73 ± 0.13	0.66 ± 0.10
ETP	0.62 ± 0.04	**0.83** ± **0.01**	0.75 ± 0.01	0.65 ± 0.02	0.61 ± 0.17	**0.84** ± **0.02**	0.73 ± 0.13	0.66 ± 0.10
Log	**0.66** ± **0.03**	**0.83** ± **0.01**	**0.76** ± **0.01**	**0.68** ± **0.03**	**0.62** ± **0.14**	0.83 ± 0.03	**0.73** ± **0.12**	**0.67** ± **0.08**

### Results on Real Neuroimaging Genetics Data

In this real data study, the genotyping data is denoted by **X**, and the imaging data is denoted by **Y**. The **u** is a vector of weights of all SNPs, and **v** is a vector of weights of all imaging markers.The canonical correlation coefficients are defined as Pearson correlation coefficient between **Xu** and **Yv**, i.e., ${({\bf{X}}{\bf{u}})}^{{\rm{{\rm T}}}}{\bf{Y}}{\bf{v}}/(\Vert {\bf{X}}{\bf{u}}\Vert \Vert {\bf{Y}}{\bf{v}}\Vert )$.

**Figure 3:**
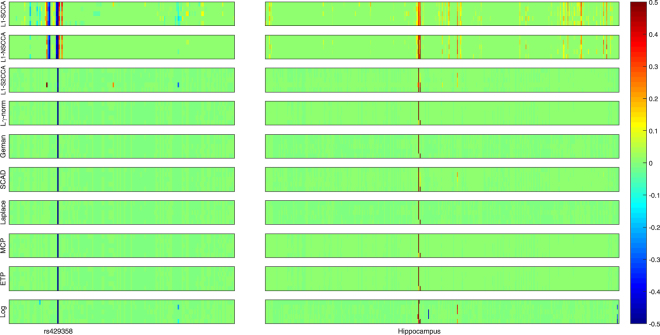
Canonical loadings estimated on real imaging genetics data. Each row corresponds to a SCCA method: (1) L1-SCCA, (2) L1-NSCCA, (3) L1-S2CCA, (4) ${\ell }_{\gamma }$-norm and so forth. For each method, the estimated ***u*** is shown on the left panel, and ***v*** is on the right one. In each individual heat map, the x-axis indicates the indices of elements in u or v (i.e., SNPs or ROIs); the y-axis indicates the indices of the cross-validation folds.

**Figure 4:**
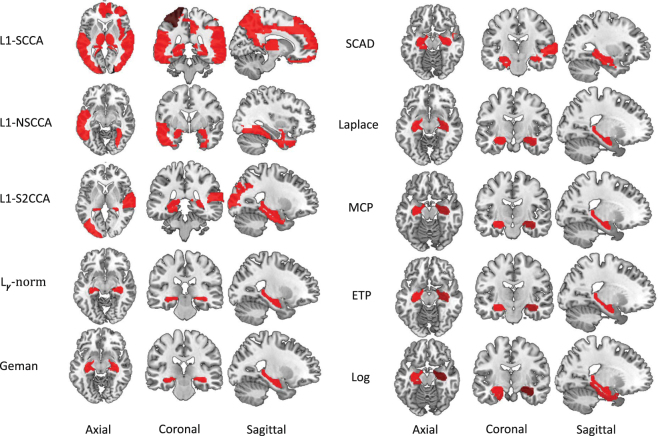
Mapping averaged canonical weight ***v***'s estimated by every SCCA method onto the brain. The left panel and right panel show five methods respectively, where each row corresponds to a SCCA method. The L1-SCCA identifies the most signals, followed by the L1-NSCCA and L1-S2CCA. All the proposed methods identify a clean signal that helps further investigation.

Figure 3 presents the heat maps regrading the canonical loadings generated from the training set. In this figure, each row shows two weights of a SCCA method, where a larger weight stands for a more importance. The weight associated with the SNPs is on the left panel, and that associated with the voxels is on the right. The proposed non-convex SCCA methods obtain very clean and sparse weights for both **u** and **v**. The largest signal on the genetic side is the *APOE* e4 SNP rs429358, which has been previously reported to be related to AD^53^. On the right panel, the largest signal for all SCCA methods comes from the hippocampus region. This is one of the most notable biomarkers as an indicator of AD, since atrophy of hippocampus has been shown to be related to brain atrophy and neuron loss measured with MRI in AD cohort^53^. In addition, the L1-S2CCA and SCAD methods identify a weak signal from the parahippocampal gyrus, which is previously reported as an early biomarker of AD^54^. On some folds, the Log method also finds out the lingual region, parahippocampal gyrus, vermis region. Interestingly, all the three regions have shown to be correlated to AD, and could be further considered as an indicating biomarker that can be observed prior to a dementia diagnosis. For example, Sjöbeck and Englund reported that molecular layer gliosis and atrophy in the vermis are clearly severer in AD patients than in the health controls^55^. This is meaningful since the non-convex SCCA methods identify the correct clue for further investigation. On this account, both L1-SCCA and L1-NSCCA are not good choices since they identify too many signals, which may misguide subsequent investigation. The figure shows that L1-S2CCA could be an alternative choice for sparse imaging genetics analysis, but it performs unstably across the five folds. And, the non-convex methods is more consistent and stable than those ${\ell }_{1}$-SCCA methods. To show the results more clearly, we map the canonical weights (averaged across 5 folds) regarding the imaging measurements from each SCCA method onto the brain in Fig. 4. The figure confirms that the L1-SCCA and L1-NSCCA find out many signals that are not sparse. The L1-S2CCA identifies fewer signals than both L1-SCCA and L1-NSCCA, but more than all these non-convex SCCA methods. All the non-convex SCCA only highlights a small region of the whole brain. This again reveals that the proposed methods have better canonical weights which reduces the effort of further investigation.

Besides, we include both training and testing correlation coefficients in Table 6, where their *mean* and *standard deviation* are shown. The training results of all methods are similar, with the Log method gains the highest value of 0.33 ± 0.03. As for the testing results, which is our primary interest, all the non-convex SCCA methods obtain better values than these ${\ell }_{1}$-SCCA methods. Besides, the difference between the training and testing performance of the proposed methods is much smaller than that of three ${\ell }_{1}$-SCCA methods. This means that the non-convex methods have better generalization performance as they are less likely to fall into overfitting issue. The result of this real imaging genetics data reveals that the proposed SCCA methods can extract more accurate and sparser canonical weights for both genetic and imaging biomarkers, and obtain higher correlation coefficients than those ${\ell }_{1}$-SCCA methods.

**Table 6:** Performance comparison on real data set. Training and testing correlation coefficients (mean ± std) of 5-fold cross-validation are shown. The best value is shown in boldface.

	L1-SCCA	L1-S2CCA	L1-NSCCA	${\ell }_{\gamma }$-norm	Geman	SCAD	Laplace	MCP	ETP	Log
Training	0.27 ± 0.01	0.29 ± 0.02	0.27 ± 0.01	0.28 ± 0.02	0.27 ± 0.02	0.29 ± 0.02	0.27 ± 0.02	0.28 ± 0.02	0.28 ± 0.02	**0.33** ± **0.03**
Testing	0.18 ± 0.04	0.25 ± 0.10	0.22 ± 0.07	0.26 ± 0.09	0.26 ± 0.10	0.27 ± 0.09	0.26 ± 0.10	0.26 ± 0.09	0.26 ± 0.09	**0.27** ± **0.11**
Training-Testing Gap	0.09	0.04	0.05	0.02	0.01	0.02	0.01	0.02	0.02	0.06

## Conclusion

We have proposed a unified non-convex SCCA model and an efficient optimization algorithm using a family of non-convex penalty functions. These penalties are concave and piecewise continuous, and thus piecewise differentiable. We approximate these non-convex penalties by an ${\ell }_{2}$ function via the local quadratic approximation (LQA)^38^. Therefore, the proposed algorithm is effective and runs fast.

We compare the non-convex methods with three state-of-the-art ${\ell }_{1}$-SCCA methods using both simulation data and real imaging genetics data. The simulation data have different ground truth structures. The results on the simulation data show that the non-convex SCCA methods identify cleaner and better canonical loadings than the three ${\ell }_{1}$-SCCA methods, i.e. L1-SCCA^25^, L1-S2CCA^32^, and L1-NSCCA^29^. These non-convex methods also recover higher correlation coefficients than ${\ell }_{1}$-SCCA methods, demonstrating that ${\ell }_{1}$-SCCA methods have suboptimal prediction capability as they may over penalize large coefficients. The results on the real data show that the proposed methods discover a pair of meaningful genetic and brain imaging biomarkers, while the ${\ell }_{1}$-SCCA methods return too many irrelevant signals. The correlation coefficients show that the non-convex SCCA methods hold better testing values. This verifies our motivation that the non-convex penalty can improve the prediction ability, and thus has better generalization capability. Obviously, the parameter *γ* plays a key role in these non-convex penalties. In the future work, we will investigate how to choose a reasonable *γ*; and explore how to incorporate structure information into the model as structure information extraction is an important task for brain imaging genetics as well as biology studies.
